# A Needle in the Arm—How Did It Get There?

**Published:** 1887-07

**Authors:** 


### A NEEDLE IN THE ARM—HOW DID IT GET THERE?

S. P. Thompson, student, writes: "In my work, as a student, in the dissecting room of the Southern Medical College, session of 1886–'7, I encountered something which I thought might prove of interest to the many readers of the RECORD. I was making a dissection of the arm of a strong muscular subject, and in raising the biceps I saw what, at first sight, I thought to be a small vein in which the blood had coagulated; but an attempt to sever it with the scalpel showed it to be a metallic substance. I then dissected it out and found it to be a common sewing-needle, the eye of which had been broken off. It was about one inch in length. The broken end was buried deeply into the brachialis anticus and the point extended up into the biceps, being at a right angle to the shaft of the humurus. The needle was very rusty, and had become encysted. How did it get there—in such a peculiar position? That is the question."

[IN the absence of the history of the case we cannot know how the needle came there, or how long it had been encysted. It may have traveled from some remote part, as instances have occurred where a needle swallowed has made its exit at some distant point of the body. A case is related in which, in an operation upon the abdomen, a probe six inches long, fell within the abdominal cavity, and being left there by the frightened physician, came out, after several months, in the posterior part of the thigh, and without any untoward symptoms. —EDITOR.]

